# Performance evaluation of the IRIS XL-220 PET/CT system, a new camera dedicated to non-human primates

**DOI:** 10.1186/s40658-022-00440-8

**Published:** 2022-02-05

**Authors:** Frédéric Boisson, Sophie Serriere, Liji Cao, Sylvie Bodard, Alessandro Pilleri, Lionel Thomas, Giancarlo Sportelli, Johnny Vercouillie, Patrick Emond, Clovis Tauber, Nicola Belcari, Jean-Luc Lefaucheur, David Brasse, Laurent Galineau

**Affiliations:** 1 Institut Pluridisciplinaire Hubert Curien, Université de Strasbourg, 23 rue du Loess, 67037 Strasbourg, France; 2 UMR7178, CNRS, 67037 Strasbourg, France; 3 UMR 1253, IBrain, Équipe Imagerie, Biomarqueurs et Thérapie, Université de Tours, Inserm, UFR Médecine, 10 boulevard Tonnellé, Bât. Planiol 4ème étage, 37000 Tours, France; 4 Département d’Imagerie Préclinique, Plateforme Scientifique et Technique Analyse des Systèmes Biologiques, Université de Tours, Tours, France; 5 Inviscan SAS, Strasbourg, France; 6 Department of Physics, University of Pisa, Largo Bruno Pontecorvo 3, 56127 Pisa, Italy

**Keywords:** PET/CT, Instrumentation, NEMA characterization, Non-human primates, Preclinical imaging, Performance evaluation

## Abstract

**Background:**

Non-human primates (NHP) are critical in biomedical research to better understand the pathophysiology of diseases and develop new therapies. Based on its translational and longitudinal abilities along with its non-invasiveness, PET/CT systems dedicated to non-human primates can play an important role for future discoveries in medical research. The aim of this study was to evaluate the performance of a new PET/CT system dedicated to NHP imaging, the IRIS XL-220 developed by Inviscan SAS. This was performed based on the National Electrical Manufacturers Association (NEMA) NU 4-2008 standard recommendations (NEMA) to characterize the spatial resolution, the scatter fraction, the sensitivity, the count rate, and the image quality of the system. Besides, the system was evaluated in real conditions with two NHP with ^18^F-FDG and (-)-[^18^F]FEOBV which targets the vesicular acetylcholine transporter, and one rat using ^18^F-FDG.

**Results:**

The full width at half maximum obtained with the 3D OSEM algorithm ranged between 0.89 and 2.11 mm in the field of view. Maximum sensitivity in the 400–620 keV and 250–750 keV energy windows were 2.37% (22 cps/kBq) and 2.81% (25 cps/kBq), respectively. The maximum noise equivalent count rate (NEC) for a rat phantom was 82 kcps at 75 MBq and 88 kcps at 75 MBq for energy window of 250–750 and 400–620 keV, respectively. For the monkey phantom, the maximum NEC was 18 kcps at 126 MBq and 19 kcps at 126 MBq for energy window of 250–750 and 400–620 keV, respectively. The IRIS XL provided an excellent quality of images in non-human primates and rats using ^18^F-FDG. The images acquired using (-)-[^18^F]FEOBV were consistent with those previously reported in non-human primates.

**Conclusions:**

Taken together, these results showed that the IRIS XL-220 is a high-resolution system well suited for PET/CT imaging in non-human primates.

## Background

Positron emission tomography (PET) is undoubtedly an essential tool for understanding the pathophysiological mechanisms related to various disorders, and for the developments of new therapeutic approaches. Based on its non-invasiveness and translational capabilities [1], it is widely used for clinical and preclinical studies in neuropsychiatry, oncology and cardiology [2–9].

While most preclinical studies are performed in rodents, working with non-human primates (NHP) is crucial for research related to the biology of diseases, and the development of new health care technologies. Preclinical research on rodents is well suited to answer basic scientific questions, but are limited by their anatomical and functional differences, especially for the brain, immune system and metabolism [10]. Even though the number of NHP used in preclinical research is estimated to be less than 1%, its impact is huge [10]. For example in brain research, NHP models offer a unique opportunity to explore complex functions based on the strong similarity between the NHP and human brains [11–14].

**Fig. 1:**
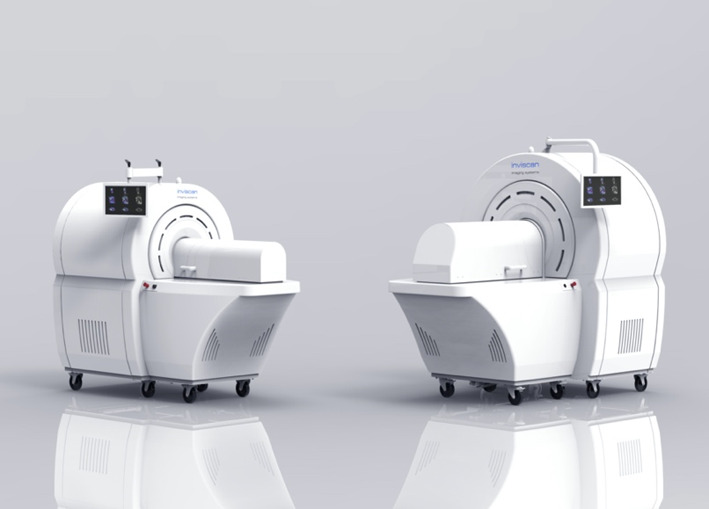
Photographs of the IRIS PET/CT (on the left-end side) and the IRIS XL-220 PET/CT (on the right-end side) systems at the core facility for preclinical imaging of the University of Tours (Département d’Imagerie Préclinique, Plateforme Scientifique et Technique Analyse des Systèmes Biologiques, Université de Tours, Tours, France)

As NHP have a definite advantage in a more translational approach to the clinical world, there is a need for preclinical PET systems dedicated to the imaging of NHP. The last 2 decades have seen the emergence of new high-performance preclinical PET systems, but almost exclusively dedicated to the imaging of small animals. To date, very few PET systems dedicated to NHP imaging have been evaluated with only one being currently on the market [15, 16]. PET systems dedicated to NHP offer better performance in terms of spatial resolution and detection efficiency compared to the use of clinical systems, specially thanks to dedicated high-resolution detectors and the more suitable gantry diameter. Here, we evaluated the IRIS XL-220 PET/CT system dedicated to NHP imaging, developed, and commercialized by the company Inviscan SAS (Fig. 1).

The present paper evaluates the performance parameters of the PET component of the IRIS XL-220 PET/CT system, based on the National Electrical Manufacturers Association (NEMA) NU 4-2008 standard recommendations [17]. The NEMA standard enables to assess the system’s characteristics, such as the spatial resolution, the scatter fraction, the sensitivity, the count rate, and the image quality. To illustrate its initial capacities for in vivo molecular imaging, we also performed a PET scan on an NHP and a rat using ^18^F-FDG widely used for PET imaging, along with another PET scan on an NHP using a radiotracer of reference specifically targeting the brain vesicular acetylcholine transporter, the (-)-[^18^F]FEOBV.

## Methods

This paper presents the characterization of the IRIS XL-220 PET system using a single detector ring leading to an axial field of view of 45 mm. Based on the same detector technology as the IRIS PET system [18], the IRIS XL-220 PET detector ring consists in 16 modules arranged in one single ring. Each module can acquire coincidences with the nine opposing modules. The PET field-of-view (FOV) has an axial coverage, and a trans-axial coverage of 45 and 170 mm, respectively. Each module consists in a 27 × 27 matrix of 1.6 × 1.6 × 16 mm^3^ Lutetium Yttrium orthosilicate Cerium-doped crystals (LYSO:Ce) with a pitch of 1.68 mm. Each matrix is directly coupled to a multi-anode PhotoMultiplier Tube (H12700A Hamamatsu Photonics K.K., Japan). Each module is completely independent from the others. The IRIS XL-220 PET data acquisition system is similar to the DAQ of the IRIS PET/CT scanners. The CT system consists of a micro-focus X-ray tube with 80 W and a flat panel CMOS detector. The CT covers a FOV of 170 mm in transaxial and 85 mm in axial direction with a single acquisition. The CT can achieve 90 μm resolution at 10% MTF and ultrafast scanning in less than 8 s.

We acquired experimental performance data following the NEMA NU 4-2008 standard (NEMA 2008). Unless specified otherwise, default data preprocessing settings were used (i.e., energy window = 250–750 keV, coincidence window = 5.2 ns) and data were processed using the Single Slice Rebinning (SSRB) algorithm as proposed by the NEMA procedure.

### Spatial resolution

According to the NEMA standard, a 0.55 MBq ^22^Na point source (1 cm^3^ cubic source, Eckert and Ziegler Isotope Products, Germany) was used to perform spatial resolution measurements. Measurements were acquired with the source located at the axial center of the FOV, and at one-fourth of the axial FOV from the center of the axial FOV, at the following radial distances from center: 0-, 5-, 10-, 15-, 25-, 50-, and 75-mm. Data were acquired for 120 s and reconstructed using the 3-dimensional ordered- subset expectation maximization (3D OSEM) algorithm with 8 iterations and 8 subsets.

The IRIS XL-220 PET system uses system matrices to reconstruct the data. The user can either choose to reconstruct the data acquired in the entire primate FOV (170 mm transverse diameter) with voxels of 0.84 × 0.84 × 0.84 mm^3^ or to reconstruct the data acquired in a transverse FOV of 80 mm in diameter, corresponding to the rodent FOV, with voxels of 0.42 × 0.42 × 0.84 mm^3^. Resolution measurements corresponding to radial distances of 0, 5, 10, 15, 25 mm were first reconstructed using the thinner voxels (within the rodent FOV) and radial distances of 50 and 75 mm were reconstructed using the larger one (within the primate FOV).

The full width at half maximum (FWHM) and full width at tenth maximum (FWTM) of the point source response function along the radial, tangential, and axial directions were determined following the NEMA suggested procedure. Results were not corrected for positron range effect or source size.

### Scatter fraction and count rate measurements

The purpose of these measurements was to evaluate the scanner performance in terms of the counting rate capability, the scatter fraction (SF), and the random coincidences rate. According to the system FOV, the protocol measurements were performed with a rat-like phantom (150-mm length, 50 mm diameter) and a monkey-like phantom (400 mm length, 100 mm diameter) made of a high-density polyethylene cylinder. Both phantoms have a cylindrical hole (3.2 mm diameter) drilled parallel to the central axis. The rat-like and monkey-like phantoms were filled with 120 MBq and 270 MBq of ^18^F-fluoride solution, respectively. Data were acquired for 1 min every 15 min using both phantoms. Data were processed without dead-time, decay, attenuation, or random counts corrections. Two energy windows of 250–750 keV and 400–620 keV were used to process data from both phantoms.

### Sensitivity

Sensitivity measurements were performed using the same source described in the spatial resolution measurements section, according to the NEMA recommendations. The source was moved along the scanner axial FOV and data were acquired with a 1 mm step for 2 min at each position.

### Image quality study

The NU4-2008 standard prescribes the use of the specific NEMA Image Quality phantom (IQP). The IQP is composed of a main phantom body that contains a fillable cylindric chamber (diameter = 30 mm × length = 30 mm) and a solid part (20 mm in length) into which five fillable rods with various diameters (1, 2, 3, 4, and 5 mm) have been drilled. A lid attached to the uniform region of the phantom supports two cold-region chambers. These regions are hollow cylinders (15 mm in length and 8 mm in inner diameter with 1 mm wall thickness), to be filled with non-radioactive water and air, respectively. The phantom was filled with 3.7 MBq of ^18^F-fluoride solution and placed in the central part of the FOV. The duration of the scan was set to 20 min. PET images were reconstructed with the 3D-OSEM algorithm (8 iterations, 8 subsets), including random, radioactive decay and dead time corrections. No attenuation correction was performed. We followed the NEMA NU-4 2008 standard for the analysis of the IQP data to compute the recovery coefficient (RC) for each rod, the spillover ratio and the signal to noise ratio.

### In vivo experiments

*Rat experiment* One male rat (262 g, 10 weeks old; Charles River) was imaged for 15 min, 1 h after an intraperitoneal injection of 18.9 MBq of 2-deoxy-2-(^18^F)fluoro-d-glucose (^18^F-FDG). Anesthesia was induced and maintained with 1.5% isoflurane in medical air with a calibrated vaporizer during the 1 h biodistribution. The rat was then euthanized for the PET acquisition with the brain centered in the system’s FOV. Data were reconstructed into a 205 × 205 × 54 three-dimensional volume by use of the iterative 3D OSEM algorithm with 8 iterations and 8 subsets. The voxel size was equal to 0.84 mm in all directions. The calibration factor was included in the normalization file and applied during the reconstruction process. PET data were fully corrected for random coincidences, radioactive decay, and dead time. No scatter correction was applied. The PET acquisition was followed by a 576 projections computed tomography (CT) at 80 kV leading to 98 s CT acquisition. This experiment was performed in accordance with European Institutes of Health Guidelines regarding the care and use of animals for experimental procedures and were approved by the Alsace Regional Ethics Committee for Animal Experimentation (approval identification: APAFIS#1531).

**Fig. 2:**
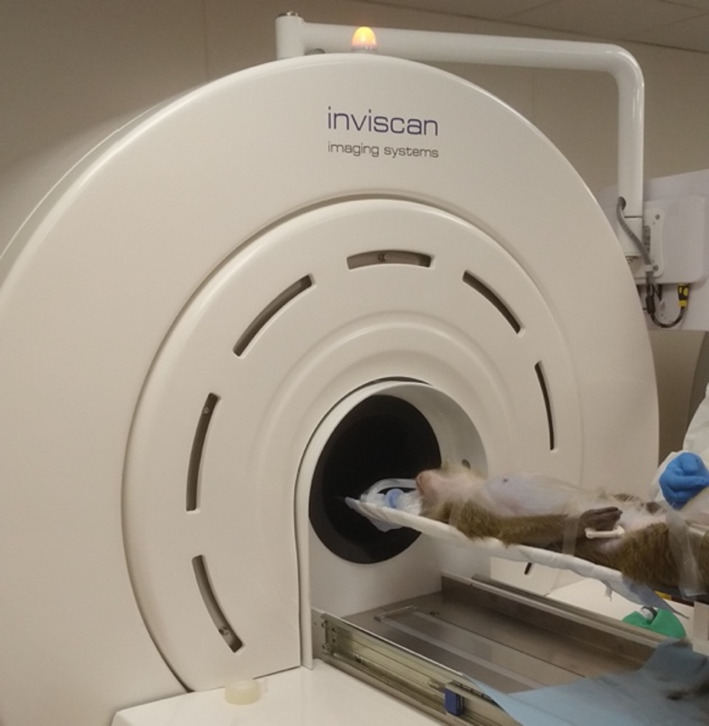
Photograph of a non-human primate experiment conducted on the IRIS XL-220 PET/CT at the core facility for preclinical imaging of the University of Tours (Département d’Imagerie Préclinique, Plateforme Scientifique et Technique Analyse des Systèmes Biologiques, Université de Tours, Tours, France)

*NHP experiments* Two NHP experiments were performed in accordance with EU guidelines (EU Directive 2010/63/EU for animal experiments) and approved by a local Ethics Committee (Comité d’Ethique en Experimentation Animale—Pays de la Loire—France—approval identification: APAFIS#27750). Two Macaca fascicularis females weighing 2.9 and 4.5 kg were used for this study (Fig. 2). Anesthesia was induced by an intramuscular injection of a mixture of ketamine/medetomidine (3 mg/kg and 125 μg/kg, respectively). Each NHP was placed on a heating blanket, and intubated. Anesthesia was then maintained using Vetflurane (1%; Coveto, France), and each NHP was placed on the bed of the system positioned on its back with the head taped to limit its movements. A catheter was inserted in the saphenous vein for the radiotracer injection. During all the experiment, the NHP were monitored for their body temperature, heart rate, respiratory rate, and oxygenation.

In a first experiment, the whole brain uptake of ^18^F-FDG (Curium, France) was evaluate using a two-bed PET and CT scans performed with the center of the field of view positioned in the center of the brain. The NHP (4.5 kg) was injected with ^18^F-FDG (85 MBq i.v.) and imaged for 30 min using a static acquisition (15 min per bed position) starting 40 min after the radiotracer injection. PET images were reconstructed with the 3D-OSEM algorithm (8 iterations, 8 subsets), including random, radioactive decay, dead time corrections, and attenuation correction. The resultant images included 90 slices with a voxel size of 0.84 mm in all directions. A two-bed CT scan was performed considering 576 projections over 360 degrees, at 80 kV and 0.9 mA with a 90 ms exposure time, leading to a total acquisition time of 104 s. The three-dimensional image was reconstructed with beam hardening correction, ring artifacts pre-correction, resulting in 1190 slices with a 160 × 160 × 160 μm voxel size.

In a second experiment, another NHP (2.9 kg) was injected intravenously with 92 MBq of [^18^F]fluoroethoxybenzovesamicol ((-)-[^18^F]FEOBV), a radiotracer of reference specifically targeting the vesicular acetylcholine transporter [19, 20]. (-)-[^18^F]FEOBV was synthesized using an adapted method of the original process [19]. The corresponding enantiomerically pure precursor of the (-)-[^18^F]FEOBV, the (-)-TEBV was purchased to ABX advanced biochemical compounds GmbH. A TRACERlab FXFN Pro (General Electric Healthcare) was used as synthesizer. [^18^F]fluoride was purchased to Curium Pharma. Sep-Pak Accell Light QMA, *t*-C18 and light *t*-C18 cartridges were purchased from Waters. High performance liquid chromatography quality control (QC) was performed on an Ultimate 3000 system equipped with a UV detector and a radioactivity detector (PET metabolite Bioscan). The column for the purification and QC Luna Phenyl Hexyl (Phenomenex) with respectively the following specifications 10µ, 4.6 × 250 mm and 5µ, 4.6 × 250 mm. In these conditions, we obtained pure (-)-[^18^F]FEOBV in 79 min with a radiochemical yield of 62.6% with a molar activity of 307.3 GBq/µmol.

Imaging was performed using the same procedure as previously described, with a 98 s CT scan performed to place the brain in the center of the field of view, followed by a PET dynamic list-mode acquisition starting 1 min before (-)-[^18^F]FEOBV injection and lasting for 121 min. PET images were reconstructed as previously described with the following defined frames: 1 × 60 s, 18 × 30 s, 11 × 60 s, 10 × 10 min corresponding to a total of 40 frames. A one bed CT scan has also been performed using the parameters previously described.

Each scan was manually registered on a brain Macaca fascicularis MRI template (www.cermep.fr/download/atlas; [20]) using PMOD v4.203 software (PMOD Technologies Ltd, Switzerland). ^18^F-FDG and (-)-[^18^F]FEOBV time activity curves were derived from regions of interest defined in the template. (-)-[^18^F]FEOBV concentrations were expressed as standardized uptake values (SUV).

## Results

### Spatial resolution

The FWHM and FWTM at axial center and at one quarter from the axial center obtained with the 3D OSEM algorithm are presented in Tables 1 and 2, respectively. Spatial resolution values presented in both Tables 1 and 2 were measured in images reconstructed considering the rodent FOV (values on the left) and the primate FOV (values on the right), respectively. The full width at half maximum obtained with the 3D OSEM algorithm ranged between 0.89 and 2.11 mm in the studied FOV. The full width at tenth maximum obtained with the same algorithm ranged between 1.66 and 4.44.

**Table 1:** FWHM and FWTM at axial center resolutions at different radial positions

Radial distance (mm)	Tangential (mm) Rodent/primate		Radial (mm) Rodent/primate		Axial (mm) Rodent/primate	
	FWHM	FWTM	FWHM	FWTM	FWHM	FWTM
0	0.96/1.22	2.26/2.22	1.08/1.07	2.34/1.96	1.52/1.1	2.44/2.00
5	0.93/1.19	2.21/2.16	1.00/1.01	2.27/1.84	1.58/1.09	2.45/1.99
10	1.03/1.09	2.29/1.98	1.09/1.08	2.38/1.96	1.67/1.12	2.46/2.04
15	1.00/1.06	2.34/1.93	1.10/1.29	2.61/2.36	1.74/1.09	2.46/1.99
25	1.14/1.39	2.68/2.54	1.36/1.52	3.11/2.77	1.47/1.05	2.42/1.92
50	1.40	2.55	1.90	3.47	0.92	1.67
75	1.51	2.75	2.11	3.85	0.91	1.66

Slice thickness (mm): 0.84

**Table 2:** FWHM and FWTM at one quarter from the axial center resolutions at different radial positions

Radial distance (mm)	Tangential (mm) Rodent/primate		Radial (mm) Rodent/primate		Axial (mm) Rodent/primate	
	FWHM	FWTM	FWHM	FWTM	FWHM	FWTM
0	0.89/1.19	2.21/2.17	1.06/1.04	2.25/1.90	1.66/1.07	4.44/1.95
5	1.06/1.10	2.29/2.00	1.01/1.00	2.23/1.83	1.64/1.06	3.52/1.93
10	0.97/1.02	2.23/1.86	1.05/1.06	2.34/1.93	1.51/1.05	3.42/1.90
15	0.99/1.04	2.29/1.90	1.06/1.27	2.45/2.31	1.32/0.97	2.42/1.77
25	1.11/1.37	2.56/2.50	1.33/1.50	3.06/2.73	1.08/0.98	2.38/1.78
50	1.33	2.42	1.90	3.46	1.11	2.02
75	1.42	2.59	2.11	3.85	1.05	1.91

Slice thickness (mm): 0.84

### Scatter fraction and count rate measurements

**Fig. 3:**
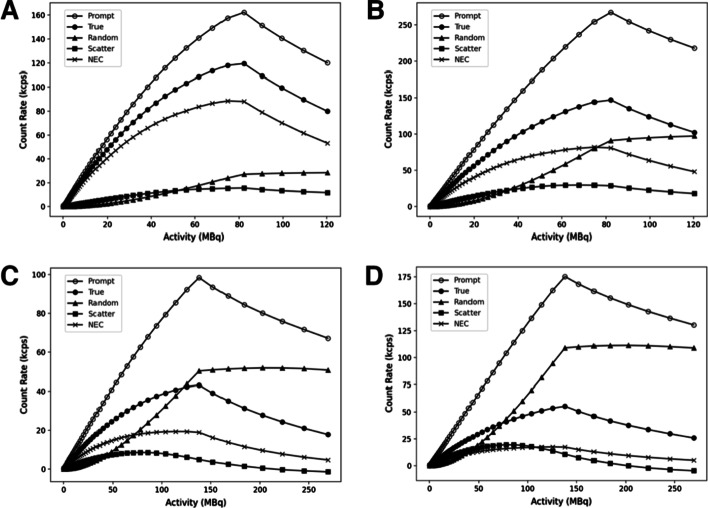
System total, true, random, and scattered event rates, and system NEC as function of the activity for the rat-like (A and B) and the monkey-like (C and D) phantoms considering the 400–620 keV and 250–750 keV energy windows, respectively

Figure 3 presents the results obtained using both the rat-like and the primate-like phantoms. For the rat phantom, the maximum NEC is 82 kcps at 75 MBq for an energy window of 250–750 keV with a scatter fraction equals to 20% at low activity. As recommended by NEMA procedure, the value of the peak true count rate is 147.04 kcps at 82 MBq in these conditions. Reducing the energy window to 400–620 keV increased the NEC to 88 kcps at 75 MBq and decreased the scatter fraction to 10%, and the peak true count rate value to 119.49 kcps at 82 MBq. For the monkey phantom, the maximum NEC is 18 kcps at 126 MBq for an energy window of 250–750 keV with a scatter fraction equals to 40% at low activity. In these conditions, the peak true count rate value is 55.24 kcps at 238 MBq. Reducing the energy window to 400–620 keV increased the NEC to 19 kcps at 126 MBq and decreased the scatter fraction to 25%, and the peak true count rate value to 43.12 kcps at 138 MBq.

### Sensitivity

**Fig. 4:**
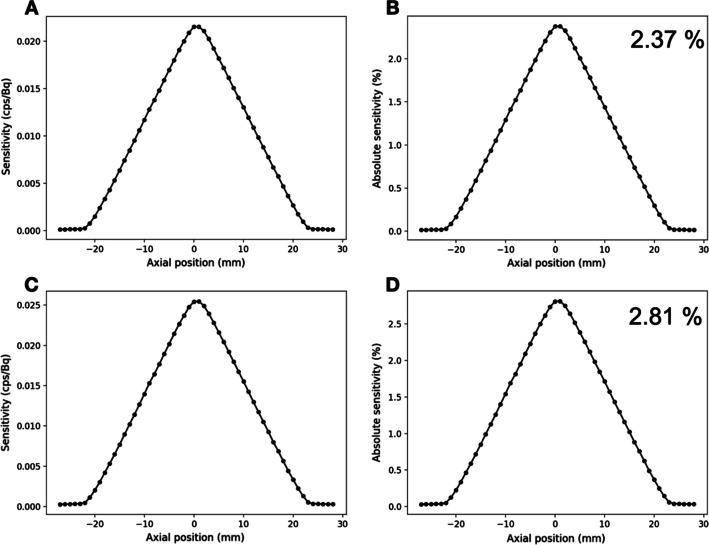
Sensitivity and absolute sensitivity profiles considering a 400–620 keV energy window (**A**, **B**) and a 250–750 keV energy window (**C**, **D**)

Figure 4 presents the sensitivity profiles along the axial axis. Maximum absolute sensitivity in the 400–620 keV and 250–750 keV energy windows were 2.37% (corresponding to 22 cps/kBq) and 2.81% (corresponding to 25 cps/kBq), respectively.

### Image quality study

The RCs are reported in Table 3. In addition, we measured a Standard Deviation of 9.58% in the uniform-activity region (Table 4), and spillover ratios for water and air were 0.17 (with 12.7% SD) and 0.05 (with 14.8% SD), respectively (Table 5).

**Table 3:** Recovery coefficient measurements obtained using the IQP

Rod diameter (mm)	Recovery coefficient	Recovery coefficient SD (%)
1	0.12	20.4
2	0.76	13.8
3	0.98	13.3
4	0.96	13.7
5	0.96	12.6

**Table 4:** Report for uniformity test

	Mean	Maximum	Minimum	%STD
Uniformity	11,924	17,700	7879	9.58

**Table 5:** Report for accuracy of corrections

Region	SOR	%STD
Water-filled cylinder	0.17	12.74
Air-filled cylinder	0.05	14.87

### In vivo experiments

**Fig. 5:**
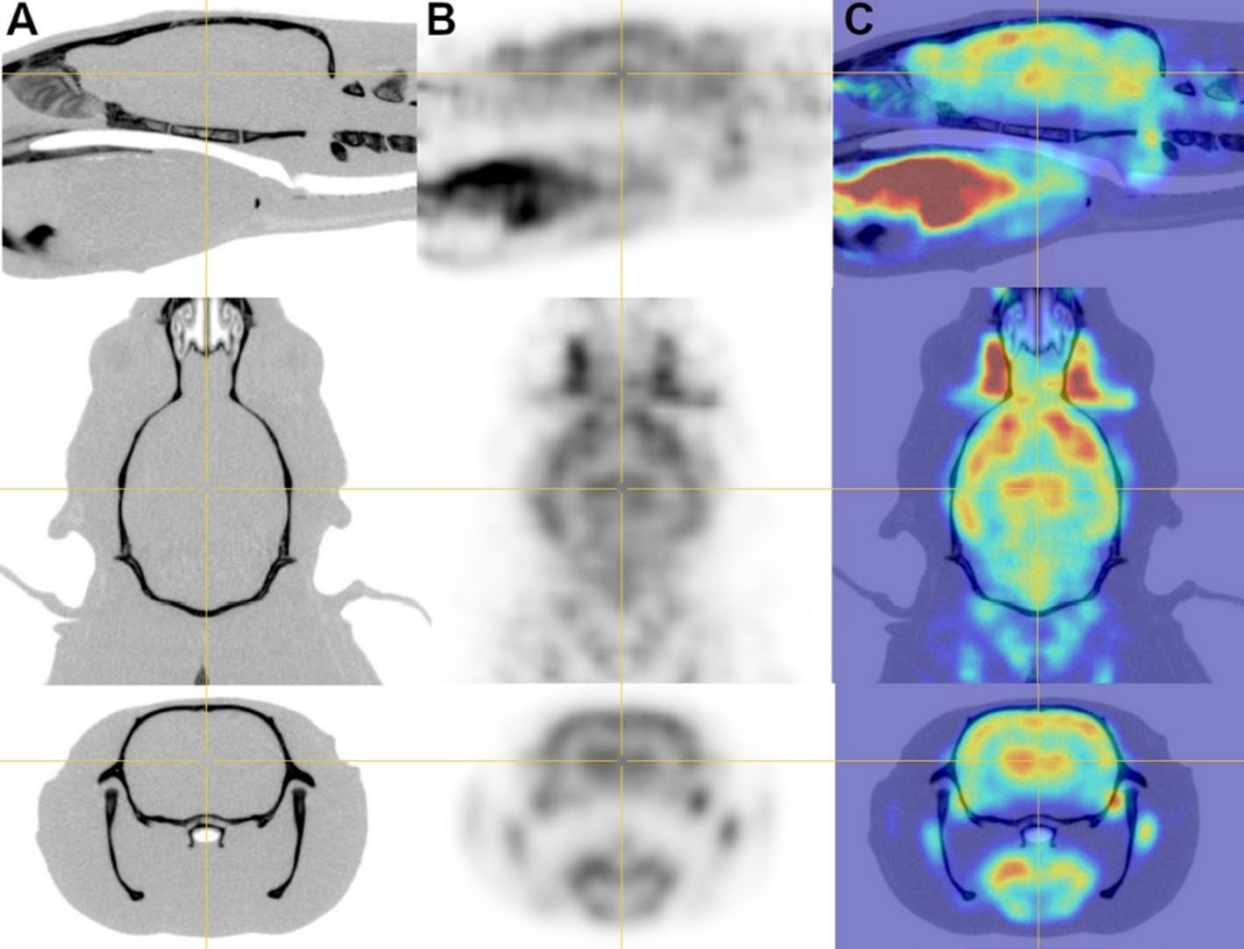
Coronal, sagittal, and axial slices of (column A) the CT, (column B) the PET and (column C) the co-registered PET/CT images of the ^18^F-FDG rat’s brain study

**Fig. 6:**
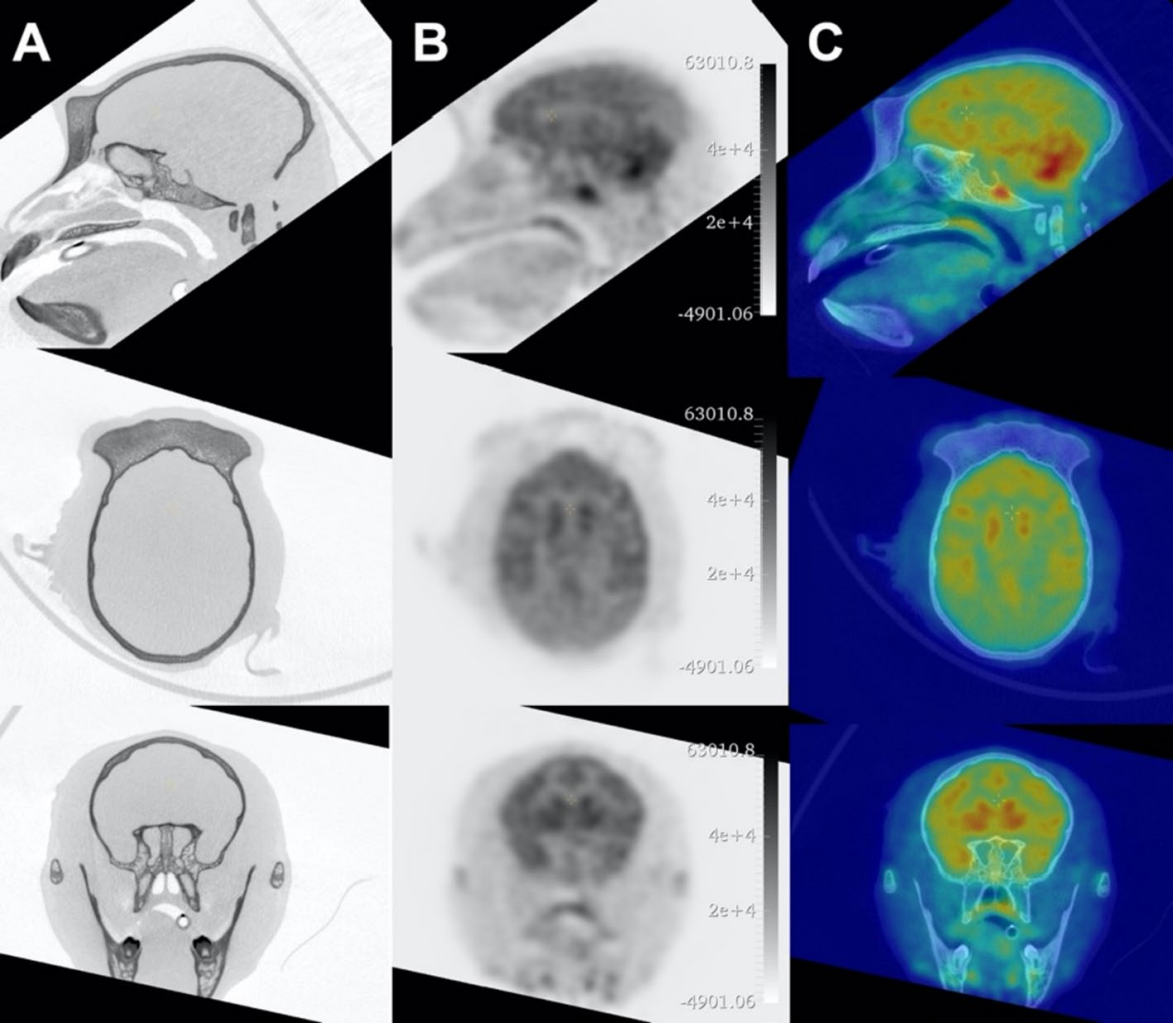
Coronal, sagittal, and axial slices of (column A) the CT, (column B) the PET, and (column C) the co-registered PET/CT images of the cerebral uptake of ^18^F-FDG of one Macaca fascicularis

The cerebral uptake of ^18^F-FDG in rats and NHP are shown in Figs. 5 and 6 along with registrations with their related CT scans. The ^18^F-FDG binding was the high in the striatum and frontal cortex compared to lower SUV observed in the occipital cortex and cerebellum (SUV of 3.0 ± 0.5, 3.0 ± 0.8, 2.7 ± 0.6, and 2.8 ± 0.6 in the striatum, frontal cortex, occipital cortex and cerebellum, respectively).

**Fig. 7:**
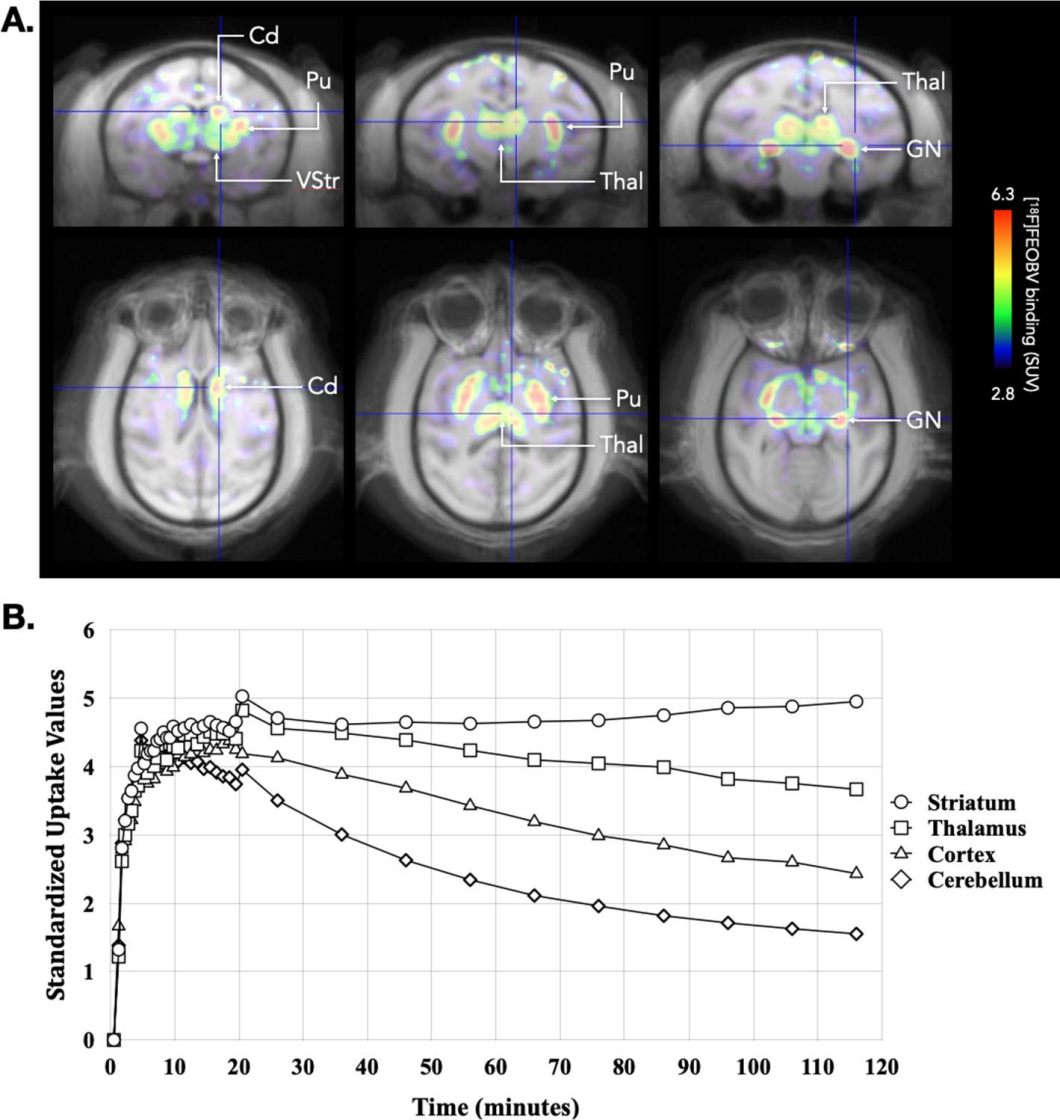
**A** Fusion between MRI and (-)-[^18^F]FEOBV SUV images (average between 80 and 120 min) showing the (-)-[^18^F]FEOBV binding in the striatum, thalamus and geniculate nuclei on representative coronal and horizontal brain slides. **B** Time-activity curves of (-)-[^18^F]FEOBV binding in the striatum, thalamus, cortex and cerebellum. *Cd* caudate, *GN* geniculate nuclei, *Pu* putamen, *Thal* thalamus, *VStr* ventral striatum

The NHP injected with (-)-[^18^F]FEOBV showed a high binding of the radiotracer in the different part of the striatum, the thalamus and geniculate nuclei compared to the lower binding observed in the cerebellum (Fig. 7).

## Discussion

In recent decades, only a few PET systems dedicated to NHPs imaging have emerged, such as the MicroPET Focus 220 Small Animal PET Scanner (formerly supplied by Concord/Siemens), or more recent systems with the LFER 150 PET/CT device (Mediso Ltd.), and the mini-Explorer displaying a long axial field of view [15, 16]. Researchers have also used clinical PET or PET systems dedicated to imaging the human brain [22, 23]. Although the clinical systems exhibit good performance and allow the imaging of NHPs, the use of dedicated scanners makes it possible to achieve better performances more in line with the subject. Like preclinical PET systems dedicated to small animals, the performance of primate systems is assessed based on the NEMA NU 4-2008 standard recommendations (NEMA).

The volumetric resolution, corresponding to the product of the radial, tangential and axial FWHM, was chosen to ensure a fair comparison with others NHP dedicated PET systems. Values of the IRIS XL-220 PET measured with the point source reconstructed with 3D-OSEM algorithm were better than the resolutions reported for both the Micro PET Focus 220 and the LFER 150 PET/CT, which were obtained using 2D FBP (1.87 vs 6.73 and 5.17 mm^3^ for the IRIS XL-220, LFER 150 PET/CT and Micro PET Focus 220, respectively). The significant differences in terms of spatial resolutions can be easily explained using different reconstruction algorithms. However, studies reported that using 2D ordered subset expectation maximization with the Focus 220 enabled to visualize rods with 1.6-mm diameter [24, 25]. Similarly, Sarnyai et al. [16] performed a small-animal Derenzo phantom study, indicating that rods with 1 mm diameter can be distinguished with the LFER 150, when using an iterative 3D reconstruction method.

In addition, it is important to mention that the spatial resolution values obtained at radial distances greater than 40 mm from the center of the FOV were measured in images reconstructed with a larger voxel size than the others. However, according to the NEMA procedure, the resolutions must be determined by linear interpolation between adjacent pixels at half (or one-tenth) the maximum value of the response function. This directly impacts the values by generating a possible bias between those obtained from images reconstructed with thinner voxels.

The absolute sensitivity obtained with the IRIS XL-220 PET was substantially higher than the one reported for the Focus 220 [24] and slightly lower than the one reported for the LFER 150 PET/CT. However, sensitivity values reported in the present paper were obtained considering a single detector ring. The absolute sensitivity of the IRIS XL-220 PET will be significantly increased in a 2 detector rings configuration.

Because a fair comparison of new preclinical PET systems based on NEMA standard evaluations has been questioned [26], we further studied the performances of the IRIS XL-220 PET system in real conditions using two distinct radiotracers. The images obtained from the ^18^F-FDG and (-)-[^18^F]FEOBV experiments on NHP were consistent with previous reports, with (-)-[^18^F]FEOBV binding corresponding to the biodistribution of the vesicular acetylcholine transporter [20, 27, 28]. This revealed that the IRIS XL-220 PET provided an excellent quality of images in NHP, but also in rats based on the spatial resolution of the system.

## Conclusion

The IRIS XL-220 PET/CT system is a high-resolution system mainly dedicated to NHP imaging. With similar performance to its rare competitors and a large transverse field of view, the IRIS XL-220 PET/CT system allows a wide range of preclinical studies. Further studies are currently conducted to assess the system’s capabilities in terms of high-throughput imaging, consisting in multiple rodents imaged simultaneously for quantification purposes. The exceptional spatial resolution coupled with the large CT FOV also makes IRIS XL the ideal system for ex-vivo studies on large samples.

## Data Availability

The datasets used and/or analysed during the current study are available from the corresponding author on reasonable request.

## Abbreviations

^18^F-FDG: 2-Deoxy-2-(^18^F)flluoro-d-glucose
3D OSEM: 3-Dimensional ordered-subset expectation maximization
(-)-[^18^F]FEOBV: (-)-[^18^F]fluoroethoxybenzovesamicol
CT: Computed tomography
FOV: Field of view
FWHM: Full width at half maximum
FWTM: Full width at tenth maximum
IQP: Image quality phantom
MRI: Magnetic resonance imaging
NEC: Noise equivalent count rate
NEMA: National Electrical Manufacturers Association
NHP: Non-human primates
PET: Positron emission tomography
QC: Quality control
RC: Recovery coefficient
SF: Scatter fraction
SOR: Spillover ratio
SSRB: Single slice rebinning
SUV: Standardized uptake values
